# Special Issue: Nanowire Field-Effect Transistor (FET)

**DOI:** 10.3390/ma13081845

**Published:** 2020-04-14

**Authors:** Natalia Seoane, Antonio García-Loureiro, Karol Kalna

**Affiliations:** 1 Centro Singular de Investigación en Tecnoloxías Intelixentes, University of Santiago de Compostela, 15782 Santiago de Compostela, Spain; 2 Nanoelectronic Devices Computational Group, College of Engineering, Swansea University, Swansea SA1 8EN, Wales, UK

**Keywords:** nanowire field-effect transistors, metal gate, material properties, fabrication, modelling, variability

## Abstract

This Special Issue looks at recent developments in the research field of Nanowire Field-Effect Transistors (NW-FETs), covering different aspects of technology, physics, and modelling of these nanoscale devices. In this summary, we present seven outstanding articles on NW-FETs by providing a brief overview of the articles’ content.

In the last years, the leading semiconductor chip manufacturing companies have introduced multi-gate, non-planar transistors into their core business with digital and analogue applications to memories, processors, and radio-frequency (RF) communication in order to achieve a larger integration on chip, increase their speed and thus data throughput and, most importantly, to reduce energy consumption. There is intense research underway to keep developing these multi-gate transistors and overcome their limitations in order to continue a transistor scaling while to further improve performance and to reduce energy consumption.

Nanowire field-effect transistors (NW-FETs) are nowadays one of the strongest contenders to replace fin field-effect transistors (FinFETs) in the following semiconductor technological nodes, because of their superior electrostatic control of the channel transport via the gate around their entire channel. This Special Issue looks at recent developments in the research field of NW-FETs. For this reason, the articles include different aspects of the physics, technology, and modelling of nanoscale NW-FETs. We present seven outstanding articles on NW-FETs by providing a brief summary of the articles’ content.

The article by Yoon et al. [1] reports on the influence of the gate and drain voltages on the charge transport properties in a zinc oxide NW-FET through temperature and voltage-dependent measurements. They found that variable-range hopping charge transport dominates the conduction in the zinc oxide NW-FET in the low temperature regime of 4 K to 100 K, whereas the thermal activation charge transport is dominant from 150 K to 300 K, diminishing the space charge-limited charge transport.

The impact of variability sources on the 10 nm gate length NW-FET is addressed in two articles. Seoane et al. [2] investigated the impact of four major sources of intrinsic variability (line-edge roughness, gate-edge roughness, metal grain granularity in a gate, and random dopants in a transistor body) on the transistor performance in digital circuits. On the other hand, Li et al. [3] analysed the effect that metal gate work function fluctuations have on the transistor DC/AC characteristics with respect to different nanoscale metal grains and the variation of aspect ratio of channel cross-sections.

The effect of the impurities in the NW-FETs was also analysed in two articles. Sano et al. [4] focused their work on the physics associated with localized impurities inside the device, describing a systematic methodology on how to treat Coulomb interaction in many body-systems when using drift-diffusion simulations. Sady et al. [5], on the other hand, studied the effect of various scattering mechanisms and nanowire cross-section shapes on electron mobility in nanoscale Si NW-FETs.

In the article by Convertino et al. [6], the authors report on the fabrication of InGaAs based on FinFETs monolithically integrated on silicon substrates, presenting results for transistors with a gate length of 90 nm and a fin width of 40 nm. These InGaAs FinFETs could potentially replace the Si FinFET technology in low-power digital and RF applications.

Finally, Lee et al. [7], in their article, reviewed the theory regarding the lowest order approximation combined with Padé approaches for the quantum-mechanical treatment of electron–phonon and phonon–phonon inelastic scattering developed within the non-equilibrium Green’s function (NEGF) formalism. The method was applied to the Si Gate-All-Around NW FET with a gate length of 13 nm. The NEGF formalism is very effective and thus a popular quantum transport technique to simulate carrier transport in very small quantum solid-state devices. The inclusion of inelastic scattering mechanisms into quantum transport techniques is very challenging, but essential to accurately account for the effect of self-heating and/or power dissipation in nanoscale semiconductor transistors because of their detrimental effect on the transistor performance and its reliability.
